# Computed tomography angiography manifestations of collateral circulations in Budd-Chiari syndrome

**DOI:** 10.3892/etm.2014.2125

**Published:** 2014-12-10

**Authors:** SHI-FENG CAI, YONG-HAO GAI, QING-WEI LIU

**Affiliations:** 1 Department of Radiology, Provincial Hospital Affiliated to Shandong University, Jinan, Shandong 250021, P.R. China; 2 Department of Ultrasound, Provincial Hospital Affiliated to Shandong University, Jinan, Shandong 250021, P.R. China

**Keywords:** Budd-Chiari syndrome, computed tomography angiography, collateral circulation

## Abstract

The aim of this study was to assess the computed tomography angiography (CTA) manifestations of collateral circulations in patients with Budd-Chiari syndrome (BCS). Eighty patients with BCS were examined by CT scan. Using the CTA images of the relevant blood vessels, including the affected hepatic veins (HVs) and inferior venae cavae (IVCs), the collateral circulations were reconstructed. In addition to obstructed HVs and IVCs, collateral circulations were found in each of the patients. The collateral circulations were classified as intrahepatic, extrahepatic and portosystemic pathways. Intrahepatic collateral pathways were further classified as the following six types: HV-accessory HV (n=51, 63.8%), HV-HV (n=6, 7.5%), HV-accessory HV plus HV (n=6, 7.5%), IVC-HV/accessory HV-HV-right atrium (n=5, 6.3%), HV-umbilical vein (n=4, 5.0%) and HV-inferior phrenic vein (n=8, 10.0%). Extrahepatic collateral pathways included IVC-lumbar-ascending lumbar-hemiazygos/azygos vein (n=80, 100.0%), IVC-left renal-ascending lumbar-hemiazygos vein (n=75, 93.8%), IVC-left renal-inferior phrenic vein (n=49, 61.3%), IVC-renal -peri-renal -superficial epigastric vein (n=26, 32.5%) and superficial epigastric vein (n=12, 15.0%) types. The CTA characteristics of each type of collateral circulation were demonstrated. In conclusion, the present study revealed that CTA is able to show the intra- and extrahepatic collateral circulations of patients with BCS, which may be useful for therapeutic planning.

## Introduction

Budd-Chiari syndrome (BCS) is a diverse group of conditions associated with obstructions of hepatic venous outflow at the level of the large hepatic vein (HV) or the extrahepatic segment of the inferior vena cava (IVC). All forms of BCS can lead to serious hemodynamic consequences and severe liver damage from intense centrilobular congestion of the liver, with ischemia, pressure necrosis and loss of parenchymal cells in the center of the liver lobule. This may be life threatening if not diagnosed and treated promptly (1,2). The most important goal in patients who refer with the suspicion of having BCS is assessment of the patency, size of the HVs and IVCs, as well as the collaterals. The obstructive process in the HVs, IVCs and the collaterals may have diverse appearances. The diagnosis of BCS predominantly depends on sonographic examination and digital subtraction angiography (DSA). However, the limitations of sonography include restriction from body habitus, intestinal gas or excessive ascites, failure to demonstrate patent veins within a congested or conversely, shrunken cirrhotic liver, failure to demonstrate retroperitoneal collaterals unless they are extremely dilated, and operator-dependency. DSA may result in vascular injury. The application of computed tomography angiography (CTA) through the use of a 64-slice spiral CT provides a noninvasive and accurate method of diagnosing BCS and manifesting the collateral circulations. The aim of this study was to illustrate the CTA characteristics of collateral circulations in BCS.

## Materials and methods

### Ethical approval

This study was approved by the Ethics Committee of the Provincial Hospital Affiliated to Shandong University (Jinan, China), and written informed consent was obtained from all patients.

### Patients

A total of 80 patients with suspected BCS following ultrasonographic examination underwent CT examination between April 2005 and December 2012 in the Provincial Hospital Affiliated to Shandong University. The patient cohort consisted of 42 males and 38 females (age range, 19–51 years; mean age, 42.58±8.92 years). The delay from the appearance of the first clinical symptoms to diagnosis ranged between 6 months and 24 years. The main clinical symptoms were right upper abdominal distention and edema of both lower extremities. Patients in whom BCS was secondary to oncothlipsis or malignant cell embolism were excluded.

### CT examination

All CT scans were performed with a 64-detector row CT scanner (Lightspeed^®^ VCT; GE Healthcare, Waukesha, WI, USA) by intravenously injecting 80 ml iopromide (Ultravist^®^ 300, 740 mg/ml) at a rate of 4 ml/sec, following 20 ml normal sodium reinjection to make optimal use of the diagnostic opacity. A dual-phase spiral CT protocol (arterial- and venous-phase) was performed. Arterial-phase imaging was performed by using bolus tracking. The data acquisition was initiated 10 sec after reaching 80 HU in the region of interest, which was positioned in the aorta at the level of the celiac artery. The portal and hepatic venous-phase acquisition times were 30 and 50 sec, respectively, after the arterial phase. The average scanning delay was 20–25 sec for the arterial phase, 55–60 sec for the portal venous phase and 75–80 sec for the hepatic venous phase following a bolus injection of contrast agent.

The CT parameters were set as follows: Voltage, 120 kV; tube current, 320 mAsec; collimation, 40 mm; pitch, 0.984; and reconstruction interval, 0.5 mm. Image reformations were conducted on a separate workstation (Syngo Volume Perfusion CT Body; Siemens Healthcare, Erlangen, Germany). Volume rendering, maximum intensity projection and multiple planar reconstruction images were acquired. Two readers (both with >10 years vascular CT experience) retrospectively evaluated the data.

## Results

Obstructed HVs and IVCs, as well as the intra- and extrahepatic collaterals, were found in each of the patients. Based on the CTA features, collateral pathways were divided into three groups: Intrahepatic, extrahepatic and portosystemic collateral pathways.

### Intrahepatic collateral circulations

#### General manifestations

CTA manifestations were consistent with the ultrasonic features described in our previous study (3). Blood from the obstructed HVs was drained to the IVC, right atrium, paraumbilical veins or inferior phrenic veins through various numbers of communicating branches of different diameters, and was then distributed to the corresponding systemic venous system. The intrahepatic collateral circulations were further classified as one of the following six types.

#### HV-accessory HV collaterals

**Figure 1:**
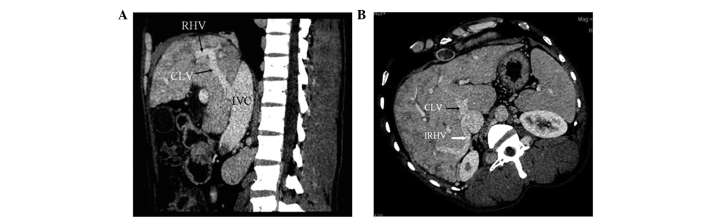
HV-accessory HV collaterals. (A) Computed tomography angiography shows segmental occlusion of the RHV. Blood drains into the IVC through the dilated CLV. (B) Blood drains into the IVC through the dilated IRHV and CLV simultaneously in another patient. HV, heptatic vein; RHV, right HV; IVC, inferior vena cava; CLV, caudate lobe vein; IRHV, inferior right HV.

In 51 patients (63.8%), CTA showed that blood from the occluded HVs was drained into the IVC through the dilated accessory HVs (Fig. 1). The accessory HVs included the caudate lobe veins and inferior right HVs. Caudate lobe veins are the largest veins draining from the caudate (Spiegel’s) lobe and flowing directly into the retrohepatic segment of the IVC (4–6). Inferior right HVs are predominantly in segment VI and in the transverse section; these veins are located at the posterior interior side of the posterior right branch of the portal vein and situated within the hepatic parenchyma in the renal impression. The inferior right HVs enter into the IVC at the level of the first portal hilum (7).

#### HV-HV collaterals

In six cases (7.5%), each patient had at least one patent HV (draining vein). The CTA manifestations were similar to those for the HV-accessory HV collateral, with the exception that the draining accessory HV was replaced by the patent HV. CTA showed that the distal region of the occluded HVs connected to the IVC through the draining veins. The lumina of the draining veins were abnormally dilated.

#### HV-accessory HV plus HV collaterals

In six cases (7.5%), CTA showed that the occluded HVs connected to the IVC by means of patent and accessory HVs (draining veins) simultaneously through communicating branches.

#### IVC-HV/accessory HV-HV-right atrium collaterals

**Figure 2:**
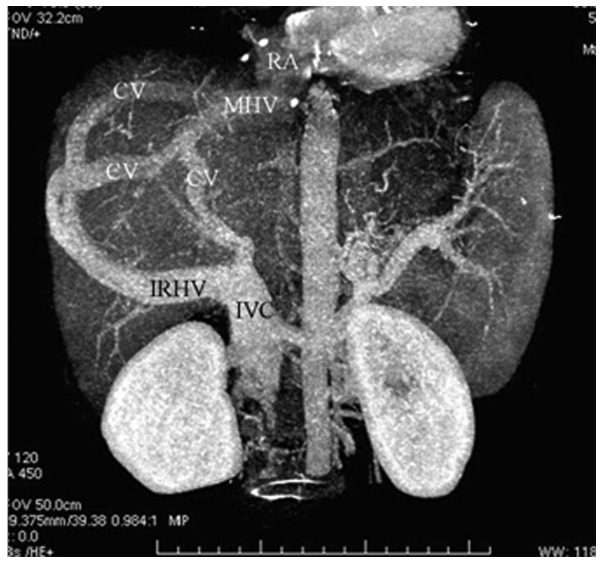
IVC-HV/accessory HV-HV-right atrium collaterals. Computed tomography angiography shows segmental occlusion of the IVC. Blood flow within the IVC reverses to the IRHV and then continues through the CVs to the MHV and then to the RA. IVC, inferior vena cava; HV, heptatic vein; IRHV, inferior right HV; CV, communicating vein; MHV, middle HV; RA, right atrium.

Segmental (occluded length >1.5 cm) or septal (septum thickness ≤1.5 cm) IVC occlusion was detected in five cases (6.3%). Reversed blood in the HV/accessory HV (inlet located below the IVC occlusion) flowed through the web-like communicating vessels, and then to the other HV (inlet located above the IVC occlusion), prior to arriving at the right atrium (Fig. 2).

#### HV-umbilical vein collaterals

**Figure 3:**
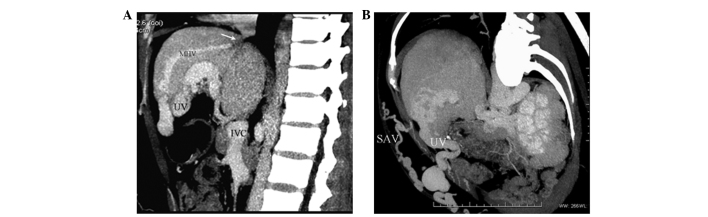
HV-UV collaterals. (A) Computed tomography angiography shows segmental occlusion (arrow) of the MHV. Blood flow in distal region of the MHV reverses to the reopened UV through communicating branches. (B) Blood from the UV enters the dilated paraumbilical vein and then the SAVs. HV, heptatic vein; MHV, middle HV; UV, umbilical vein; SAV, anterior abdominal-wall vein.

In four cases (5.0%), CTA showed segmental obstructions of three HVs. Blood flow in the distal region of the occluded HVs reversed to reopened paraumbilical veins through communicating branches, anastomosing with portal venous flow. Blood then entered the dilated paraumbilical vein and the anterior abdominal-wall veins (Fig. 3).

#### HV-inferior phrenic vein collaterals

**Figure 4:**
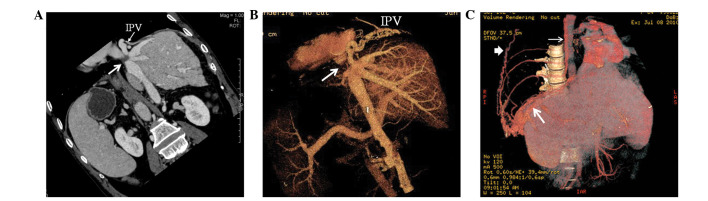
HV-IPV collaterals. (A) CTA shows segmental occlusions of both the IVC and HVs (thick arrow). The dilated IPV can also be seen (thin arrow). (B) Blood in the occluded HVs flows into the IPV. (C) CTA in another patient shows that blood in the occluded HVs flows retrogradely through the web-like IPV (thick arrow), the intercostal veins and then into the azygos vein (thin arrow). Blood from the IPV is also drained by a varicosity over the chest wall in this case (arrow head). HV, hepatic vein; CTA, computed tomography angiography; IVC, inferior vena cava; IPV, inferior phrenic vein.

In eight cases (10.0%), three HVs of this type were obstructed to different degrees without dilated accessory HVs. Blood in the obstructed HVs flowed through the inferior phrenic, intercostal and retroperitoneal veins, and then into the systemic venous system (Fig. 4).

### Extrahepatic collateral circulations

#### General manifestations

Extrahepatic collaterals resulted from an obstruction in the IVC. The extrahepatic collateral circulations were further classified as one of the following five types.

#### IVC-lumbar vein-ascending lumbar vein-azygos/hemiazygos vein collaterals

**Figure 5:**
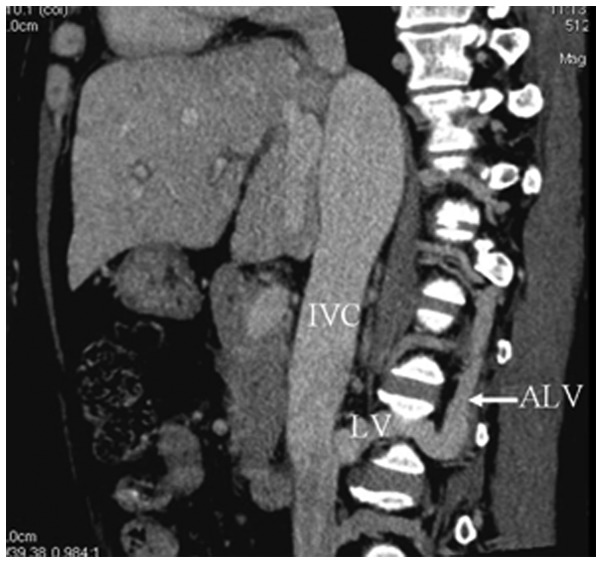
IVC-LV-ALV-azygos/hemiazygos vein collaterals. Computed tomography angiography shows membranous occlusion of the IVC. Venous flow within the IVC reverses to the LV and then continues through the ALVs, anastomosing with the azygos/hemiazygos vein system. IVC, inferior vena cava; LV, lumbar vein; ALV, ascending LV.

Collateral circulations were shown with CTA in all patients (100.0%). Venous flow within the IVC reversed to the lumbar vein and then continued through the ascending lumbar veins, anastomosing with the azygos/hemiazygos vein system (Fig. 5).

#### IVC-left renal vein-ascending lumbar vein-hemiazygos vein collaterals

**Figure 6:**
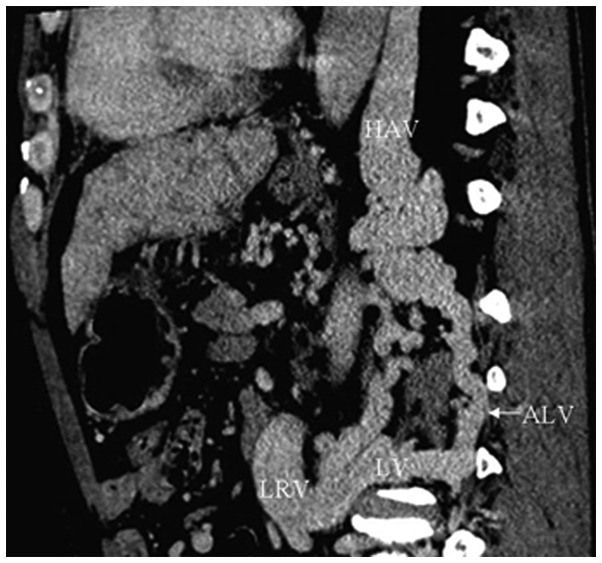
IVC-left renal vein-ALV-hemiazygos vein collaterals. Computed tomography angiography shows the connection of the dilated left renal vein with the apparently dilated and tortuous HAV by means of the LV and ALV. IVC, inferior vena cava; LV, lumbar vein; ALV, ascending LV; HAV, hemiazygos vein.

In 75 cases (93.8%), CTA showed dilated left renal veins connecting with apparently dilated and tortuous hemiazygos veins by means of the lumbar vein and ascending lumbar vein (Fig. 6).

#### IVC-left renal vein-left inferior phrenic vein collaterals

**Figure 7:**
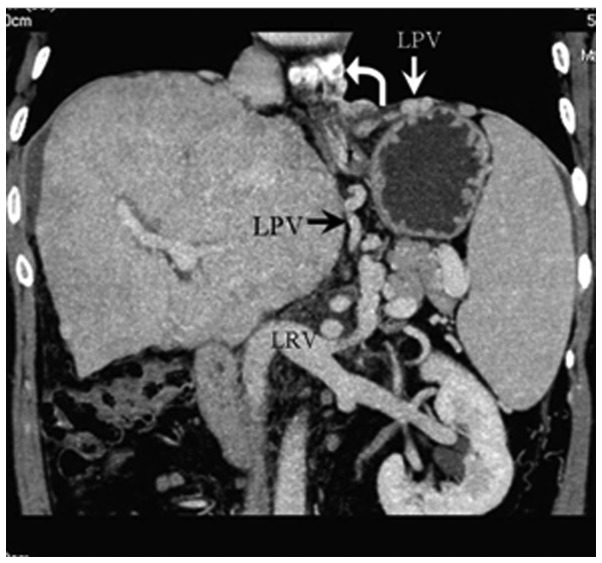
IVC-left renal vein-LPV circulation. Computed tomography angiography shows the dilated left renal vein communicating with the LPV. Tortuous blood vessels in the cardiophrenic angle can also be observed (curved arrow). IVC, inferior vena cava; LPV, left inferior phrenic vein.

In 49 cases (61.3%), CTA showed blood flow from the IVC reversed to the dilated left renal vein and then to the left inferior phrenic vein. Tortuous blood vessels in the cardiophrenic angle were also found in five patients simultaneously (Fig. 7).

#### IVC-renal vein-peri-renal vein-superficial epigastric vein collaterals

**Figure 8:**
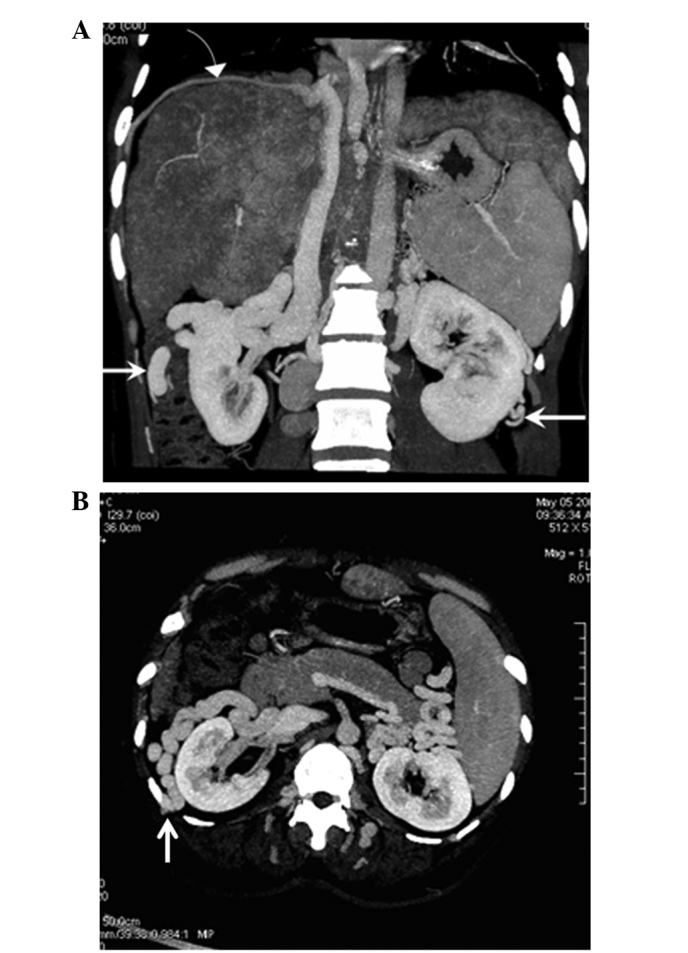
IVC-renal vein-peri-renal vein-superficial epigastric vein collaterals. (A) CTA shows bilateral renal veins communicating with peri-renal veins (straight arrow). The dilated right inferior phrenic vein can also be observed (curved arrow). (B) The transverse section of the CTA shows the distended and tortuous right peri-renal veins anastomosing with the superficial epigastric vein (arrow). IVC, inferior vena cava; CTA, computed tomography angiography.

In 26 cases (32.5%), CTA showed communication between bilateral renal veins and peri-renal veins that were evidently distended and anastomosed with the superficial epigastric vein (Fig. 8).

#### Superficial epigastric vein collaterals

**Figure 9:**
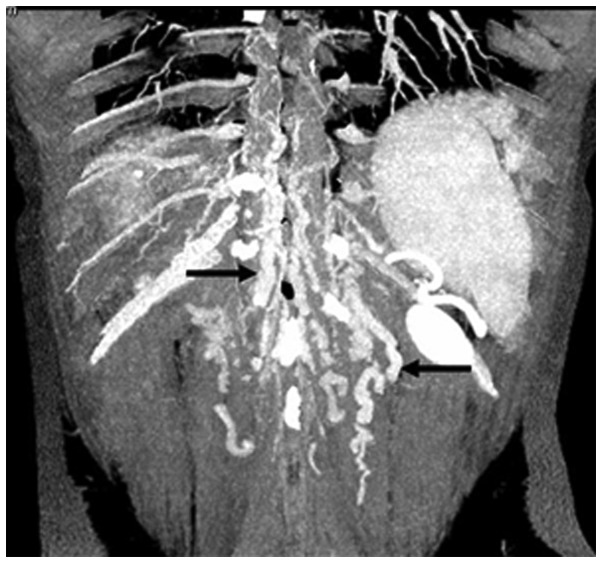
Superficial epigastric vein collaterals. Computed tomography angiography shows the expanded superficial epigastric vein collaterals (arrows).

In 12 cases (15.0%). CTA showed that the dilated inferior epigastric vein, arising from the external iliac vein, anastomosed with the superior epigastric vein above the umbilicus and with the internal mammary vein to reach the subclavian vein (Fig. 9).

Eighty patients with intrahepatic collateral circulations were combined with one or more extrahepatic collateral circulations in this group.

### Portosystemic shunts

**Figure 10:**
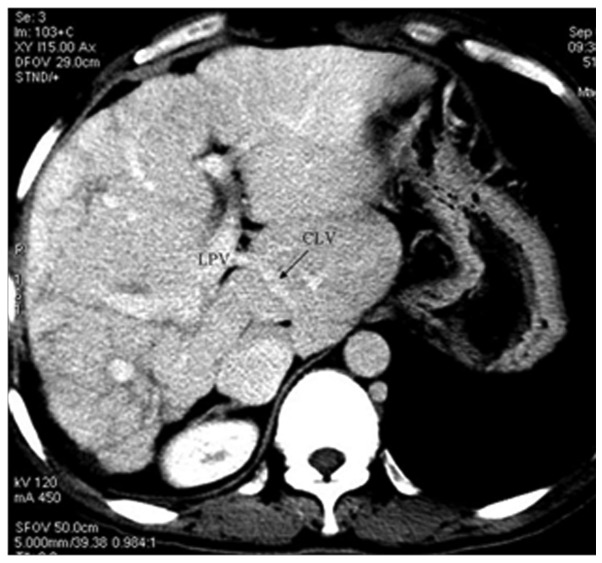
Portosystemic shunts. Computed tomography angiography shows the LPV connecting with the inferior vena cava via the patent CLV. LPV, left portal vein; CLV, caudate lobe vein.

Spontaneous portosystemic shunts were found in 16 cases. CTA showed portal veins connected with patent hepatic, accessory hepatic or left renal veins. Among the 16 patients, portal veins communicated with inferior HVs, caudate lobe veins and left HVs in eight, four and two cases, respectively, and communication with left renal veins through splenic veins occurred in two cases (Fig. 10). Blood within the portal veins flowed into the systemic venous system via the corresponding patent vessels. Hepatic cirrhosis was simultaneously shown on CT scan. In sixteen patients, spontaneous portosystemic shunts were combined with one or more type of intra- or extrahepatic collateral circulations.

## Discussion

Imaging of the HVs on enhancement CT examination occurs as of result of the back-streaming of contrast medium from the hepatic sinusoid into different-grade branches of the HVs. The enhancement of HVs is affected not only by the dose, concentration and injected flow rate of the contrast medium, but also by the delayed scan time. Normally, image formation of the HVs is more difficult than that of the hepatic arteries and portal veins. In patients with BCS, the time interval to the peak enhancement of the HV is prolonged due to the disordered hepatic blood flow that occurs as a result of obstructions in the HV and/or IVC; therefore, in the present study, a time interval of 75–80 sec after injection was selected. As a result, the afflictions of the HVs and IVCs, as well as the collateral circulations, were satisfactorily displayed.

Diverse compensatory intra- and extrahepatic circulation collaterals are demonstrated with CTA due to the different obstruction positions and lengths of the HVs and IVCs. According to Gray’s Anatomy (8), in addition to the main blood draining vessels, including the left, middle and right HVs of the liver, there is another inferior group of small-diameter HVs, which drain blood from the inferior portion of the right and caudal lobe of the liver. The HVs in this inferior group are also known as accessory HVs. Intrahepatic collaterals can be found between HVs and accessory HVs, HVs and HVs or HVs and portal veins in normal subjects (9,10). With the development of obstructions in the HVs, blood from the obstructed HVs can be compensatorily drained through the dilated collateral circulations (11,12). The intrahepatic blood drainage route is the shortest pathway with the lowest resistance. Furthermore, the IVC-HV/accessory HV-HV-right atrium collateral can efficiently compensate the outflow of the IVC to the heart, as shown in the literature (13,14) and in our previous study (15). The pathology underlying this type of collateral is that the blood pressure below the obstruction of the IVC exceeds that of the HVs, and blood from the IVC reverses through the intrahepatic collaterals to the right atrium.

The diagnosis of the HV-umbilical vein and HV-inferior phrenic vein drainage types predominantly depends on DSA, and reports of CTA descriptions are rare. The results of the present study show that CTA can clearly demonstrate not only the afflictions of the HVs but also the intra- or extrahepatic collateral circulations.

In the event of chronic IVC occlusion, collateral pathways must develop to maintain venous drainage to the right atrium. As a result, blood from the IVC may flow retrogradely through the lumbar vein-ascending lumbar vein-azygos vein and then through the hemiazygos vein into the right atrium. This is the most common extrahepatic approach and was demonstrated in all 80 patients in this group.

Anatomically, the left renal vein receives blood from multiple vessels, including the inferior phrenic, renal capsule, adrenal gland and gonadal veins; however, there is still an anatomical shunt, in which the left renal vein and the ascending lumbar, hemiazygos and vertebral veins are directly or indirectly connected through pre-existing lumbar veins (16). Ordinarily, the lumen of this shunt is minimal and without any physiological significance. In cases of IVC obstruction, as the IVC hypertension passes on to the left renal vein, the shunt may dilate and become another main blood-draining route for the IVC. The presence of such a pathway may result in para-aortic dilated and tortuous vessels connecting with the left renal vein on CTA. These collaterals were found in most of the patients (93.8%) in the present study.

As noted previously (16), the left inferior phrenic vein has two branches, one ending in the left renal vein and the other ending in the IVC. When the left renal vein is patent, a direct connection with the inferior phrenic vein may occur. As a result of the pericardiacophrenic vein, a tributary of the left brachiocephalic vein, which has diaphragmatic branches that anastomose with the inferior phrenic vein, hypertension due to IVC obstruction may result in pericardiacophrenic varices, which can appear as a vascular mass in the left cardiophrenic angle and be traced upward along the left border of the left ventricle with CTA.

Unlike the left inferior phrenic vein, the right inferior phrenic vein usually drains directly into the IVC (16); therefore, blood in the right renal vein cannot be drained by the right inferior phrenic vein. By contrast, blood flow from the right renal vein can be drained into the right atrium via renal -peri-renal-superficial epigastric venous collateral pathways, which can be clearly observed with CTA. As mentioned above, venous pressure of both renal veins can be effectively relieved by shunting blood via the collaterals, thus preserving renal function.

The superficial epigastric collateral found in the patients in the present study is another blood-draining pattern arising from an obstructed IVC. Although the collaterals of the superficial epigastric veins may be manifested on physical examination, they can be observed more clearly with CTA.

It has been reported that portosystemic connections exist both in healthy individuals and in normal individuals following mortality (17,18). In normal circumstances, there is likely to be no blood flowing through these connections due to the balanced blood pressure in the portal vein and venous system; however, when portal hypertension exists in BCS, the connections expand and shunt blood from the portal vein to the venous system, which may effectively relieve the portal vein pressure. This may therefore decrease the hepatic sinusoidal pressure and ameliorate liver function (19). The identification of spontaneous shunts using CTA may be useful in estimating the shunting capacity of blood prior to surgery.

Therapeutic strategies for BCS are based on imaging, which can demonstrate the lesions and collateral circulations of obstructed IVCs and HVs prior to surgery. The results of the present study show that the application of CTA on patients with BCS can accurately demonstrate lesions of the IVCs and HVs, as well as intra- and extrahepatic collateral circulations, which may be beneficial for therapeutic planning.
